# Association between single and mixed exposure to polycyclic aromatic hydrocarbons and biological aging

**DOI:** 10.3389/fpubh.2024.1379252

**Published:** 2024-06-05

**Authors:** Zuqiang Fu, Xianli Zhang, Chunyu Zhong, Zhe Gao, Qing Yan

**Affiliations:** ^1^School of Public Health, Southeast University, Nanjing, Jiangsu, China; ^2^Department of Neurosurgery, Children's Hospital of Nanjing Medical University, Nanjing, Jiangsu, China

**Keywords:** PAH, aging, mixed exposure, NHANES, association study

## Abstract

**Background:**

Aging is one of the most important public health issues. Previous studies on the factors affecting aging focused on genetics and lifestyle, but the association between polycyclic aromatic hydrocarbons (PAHs) and aging is still unclear.

**Methods:**

This study utilized data from the National Health and Nutrition Examination Survey (NHANES) 2003–2010. A total of 8,100 participants was used to construct the biological age predictors by using recent advanced algorithms Klemera–Doubal method (KDM) and Mahalanobis distance. Two biological aging indexes, recorded as KDM-BA acceleration and PhenoAge acceleration, were used to investigate the relationship between single PAHs and biological age using a multiple linear regression analysis, and a weighted quantile sum (WQS) model was constructed to explore the mixed effects of PAHs on biological age. Finally, we constructed the restricted cubic spline (RCS) model to assess the non-linear relationship between PAHs and biological age.

**Results:**

Exposure to PAHs was associated with PhenoAge acceleration. Each unit increase in the log10-transformed level of 1-naphthol, 2-naphthol, and 2-fluorene was associated with a 0.173 (95% CI: 0.085, 0.261), 0.310 (95% CI: 0.182, 0.438), and 0.454 (95% CI: 0.309, 0.598) -year increase in PhenoAge acceleration, respectively (all corrected *P* < 0.05). The urinary PAH mixture was relevant to KDM-BA acceleration (β = 0.13, 95% CI: 0, 0.26, *P* = 0.048) and PhenoAge acceleration (β = 0.59, 95% CI: 0.47, 0.70, *P* < 0.001), and 2-naphthol had the highest weight in the weighted quantile sum (WQS) regression. The RCS analyses showed a non-linear association between 2-naphthol and 2-fluorene with KDM-BA acceleration (all *P* < 0.05) in addition to a non-linear association between 1-naphthol, 2-naphthol, 3-fluorene, 2-fluorene, and 1-pyrene with PhenoAge acceleration (all *P* < 0.05).

**Conclusion:**

Exposure to mixed PAHs is associated with increased aging, with 2-naphthol being a key component of PAHs associated with aging. This study has identified risk factors in terms of PAH components for aging.

## 1 Introduction

Aging refers to the gradual physiological changes that occur in an organism, leading to a decline in biological function and a decreased ability of the organism to adapt to metabolic stress. Age-related disease burdens account for approximately 51.3% of the total disease burden globally (1). Biological age is a predictor of aging and indicates the likelihood of people developing chronic diseases that can be separated from chronological age. In recent years, heredity and lifestyle factors have been reported to be related to biological age. However, research on the effect of environmental chemicals on biological age remains unclear.

To date, there are only a few reports on the association between environmental chemicals and biological age. Li et al. observed that exposure to polycyclic aromatic hydrocarbons (PAHs) may be associated with an adverse impact on DNA methylation and aging (2). Campisi et al. found that PAHs may accelerate biological aging as indicated by a series of detected indicators, including lymphocyte DNA methylation age (DNAmAge), telomere length (TL), and early nuclear DNA (nDNA), which are hallmarks of non-mitotic and mitotic cellular aging, and mitochondrial DNA copy number (mtDNAcn) (3). Pavanello et al. observed that everyday life exposure to PAHs may increase the aging risk by reducing leukocyte telomere length (LTL) and mitochondrial DNA copy number (LmtDNAcn) (4). Li et al. found that exposure to PAHs might be associated with an adverse impact on human aging and epigenetic alterations in Chinese populations (2). Vriens et al. found that metals, organohalogens, and perfluorinated compounds were associated with mitochondrial DNA content and leukocyte telomere length, two putative biomarkers of aging (5). Recently, metals have been reported to be associated with accelerated aging (6). These epidemiological studies suggested an association between environmental chemicals and aging. However, the above biomarkers related to aging, including DNA methylation aging, mitochondrial DNA content, and telomere length, may have deficiencies. In recent years, the biological age algorithm that combines standard clinical parameters has been proven to be one of the most accurate algorithms for predicting morbidity and mortality (7).

The present study obtained 12 clinical indicators from the National Health and Nutrition Examination Survey (NHANES) research subjects and calculated two biological age indicators based on the Klemera–Doubla Method—Biological Age (KDM-BA) and the PhenoAge algorithms. We first explored the associations between exposure to mixed PAHs and biological age through a weighted quantile sum (WQS) regression model. In addition, we also investigated the dose–response curve of the selected PAH components and biological age through the restricted cubic splines (RCS) method.

## 2 Methods

### 2.1 Study population

**Figure 1:**
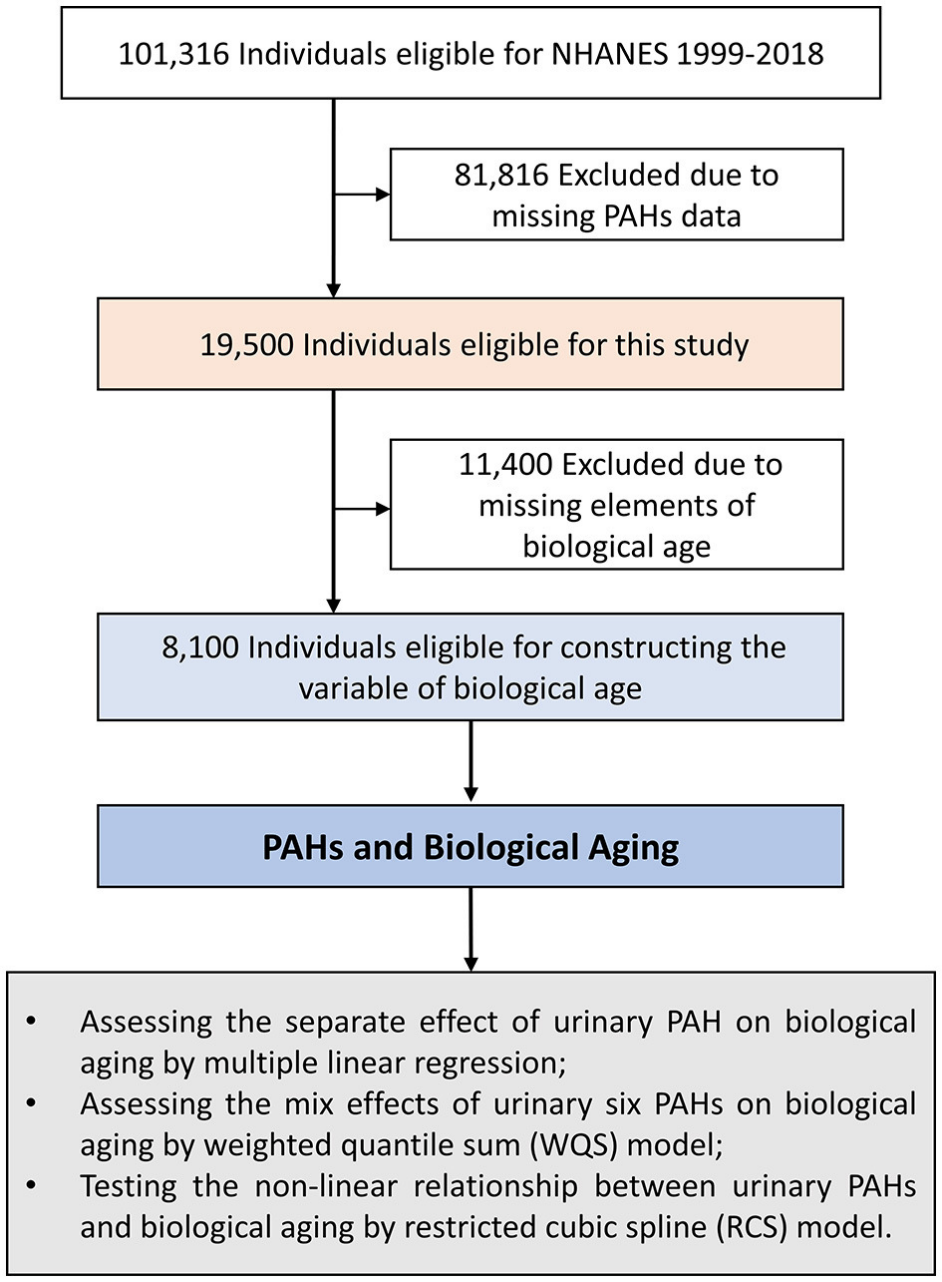
Flow chart of study population selection. NHANES, National Health and Nutrition Examination Survey; PAH, polycyclic aromatic hydrocarbon; WQS, weighted quantile sum; RCS, restricted cubic spline.

This study extracted data from NHANES 2003–2010. NHANES was a large-scale, cross-sectional survey performed by the U.S. Centers for Disease Control and Prevention, which used a multistage probability sampling design to collect nationally representative health information from non-institutionalized US civilians (8). The survey details regarding its protocol, design, operation, and quality controls have been elaborated previously (9–11). The NHANES items were approved by the research ethics committee of the Centers for Disease Control and Prevention, National Center for Health Statistics, and all participants provided informed consent forms. Data from four cycles of the NHANES survey (2003–2004, 2005–2006, 2007–2008, and 2009–2010) were used for this study. Of these populations, first, we included subjects in whom PAHs were detected (*n* = 19,500). Then, their clinical biochemical indicators were obtained, and their biological age was constructed according to the algorithm. Thereafter, persons with missing information about their biological age were excluded (*n* = 11,400). Eventually, a total of 8,100 participants were included in the subsequent analysis (Figure 1).

### 2.2 Measurement of biological age and age accelerations

We used the data available in the NAHNES public database to construct KDM-BA and PhenoAge using blood chemistry-derived measurement methods, which are among the best-validated algorithms to present biological age (12–14). In brief, KDM-BA is calculated based on the following 12 indicators: albumin (g/L), alkaline phosphatase (μ/L), C-reactive protein (natural logarithmic transformation, mg/dL), total cholesterol (mg/dL), creatinine (natural logarithmic transformation, mg/dL), glycated hemoglobin (HbA1C, %), systolic blood pressure (mmHg), blood urea nitrogen (mg/dL), uric acid (mg/dL), lymphocyte percent, mean cell volume (fl), and white blood cell count (1,000 cells/μL). PhenoAge is calculated based on eight blood chemical indicators, seven of which overlap with the indicators of KDM-BA (albumin, alkaline phosphatase, creatinine, HbA1C, mean cell volume, white blood cell count, and lymphocyte percent) and red cell distribution width according to the previous study (15). Next, we conducted KDM-BA accelerations and PhenoAge accelerations via the divergence of KDM-BA and PhenoAge from chronological age, where values >0 indicate accelerated aging and values ≤ 0 represent an equal or lower risk (physiological aging). The aging indicators were calculated with the “BioAge” R package (15, 16). The package is available on GitHub (http://github.com/dayoonkwon/BioAge) and is licensed under the GNU General Public License v3.0.

### 2.3 Assessment of urinary polycyclic aromatic hydrocarbons

Urinary PAHs were measured in a randomly sampled subpopulation of participants aged 6 years and older using capillary gas chromatography combined with high-resolution mass spectrometry and were reported as ng/L urine (17). For each sample, values below the lower limit of detection (LLD) were defined as the LLD divided by the square root of 2. Urine specimens were processed, stored, and shipped to the Division of Laboratory Sciences, National Center for Environmental Health, and Centers for Disease Control and Prevention, for analysis. The measurement and assessment of urinary PAHs involve the enzymatic hydrolysis of glucuronidated/sulfated OH-PAH metabolites in urine, extraction by online solid phase extraction, and separation and quantification using isotope dilution high-performance liquid chromatography-tandem mass spectrometry (online SPE-HPLC-MS/MS). All procedures for sample handling, transportation, storage, and other processes were performed strictly in accordance with laboratory standards. The detailed content can be reviewed on the NHANES website (https://wwwn.cdc.gov/nchs/nhanes/default.aspx) and in the NHANES Laboratory Procedure Manual (https://wwwn.cdc.gov/nchs/data/nhanes/2013-2014/labmethods/PAH_H_MET_Aromatic_Hydrocarbons.pdf). In this study, we included six PAH components for the main analyses: 1-naphthol, 2-naphthol, 3-fluorene, 2-fluorene, 1-phenanthrene, and 1-pyrene. The distribution of these six PAH components is presented in Supplementary Table S1.

### 2.4 Assessment of variates

Covariates were available from the questionnaires and laboratory examinations in the NHANES dataset. In this study, we included the following variables in the multivariate analysis. Details were as follows: age (continuous and year), sex (male and female), race (Mexican American, Other Hispanic, Non-Hispanic white, Non-Hispanic Black, and other race), education level (high school or less, some college, college graduate or above, and missing), activity (no or low, moderate, vigorous, and missing), body mass index (< 25 kg/m^2^, 25–29.9 kg/m^2^, ≥30 kg/m^2^, and missing), poverty income ratio (< 1, ≥1, and missing), serum cotinine (< LOD, LOD-10, >10, and missing), alcohol consumption (no, yes, and missing), creatinine (continuous and mg/dL), NHANES cycle (2003–2004, 2005–2006, 2007–2008, and 2009–2010), diabetes (yes and no), and hypertension (yes, no, and missing).

In addition, the detailed assessment of the above variables was as follows: the low physical activity group was defined as those with no reported leisure-time physical activity; the moderate activity group was defined as self-reported metabolic equivalents ranging from 3 to 6 of five or more times per week; and the vigorous activity group was defined as self-reported metabolic equivalents >6 of three or more times per week (18). BMI was calculated as weight in kg divided by height in meters squared and classified as normal (< 25 kg/m^2^), overweight (25–29.9 kg/m^2^), and obese (≥30 kg/m^2^) according to the standard Centers for Disease Control and Prevention classifications (19). The poverty income ratio (PIR) was defined as the ratio of family income to the poverty threshold (< 1 or ≥1) (19). Serum cotinine, a key marker of smoking exposure, was divided into less than the limit of detection (LOD), LOD to 10 ng/mL, and LOD more than 10 ng/mL (20). Information on alcohol consumption was obtained through the questionnaire: “Have you had at least 12 drinks of any type of alcoholic beverage?” The mean amounts were 12 oz. of beer, one 4 oz. glass of wine, or one ounce of liquor. The answers were “no” and “yes.”

### 2.5 Statistical analysis

In this study, continuous variables are shown as mean ± standard deviation (SD) and were analyzed with one-way ANOVA tests, while categorical variables are presented as numbers and their proportions and were analyzed with Chi-squared tests. The regression coefficient and corresponding 95% confidence intervals (CIs) were calculated using multiple linear regression analyses to investigate the associations between each unit increase in the log 10-transformed level of urinary PAHs and biological age after adjusting for age, sex, race, education level, activity, BMI, PIR, smoking status, alcohol consumption, urinary creatinine, NHANES cycle, diabetes, and hypertension. Subgroup analysis was used to evaluate the effect of positive PAHs on the age (< 60, ≥60 years) and sex groups. The above analyses were conducted with Stata version 15.1 (Stata Corp.). To investigate the comprehensive impact of mixed exposure to PAHs and to evaluate the contribution of individual PAHs, we adopted a “mixed” method based on the WQS regression analysis, which aims to evaluate the comprehensive and discrete effects of predictive factors for multiple PAH components in high-dimensional mixed backgrounds. Models were adjusted for age, sex, race, education level, activity, BMI, PIR, smoking status, alcohol consumption, urinary creatinine, NHANES cycle, diabetes, and hypertension. The weight estimation was calculated from 10,000 bootstrap samples. The random seed was set to 2023. The weight was limited to a sum of 1 and varied between 0 and 1 for comparison. To better evaluate the relationships between PAHs and two biological age indices (KDM-BA acceleration and PhenoAge acceleration), we used the restricted cubic spline (RCS) algorithm to assess their possible non-linear association in the multivariable-adjusted model. Then, we adjusted three knots to fit the RCS curve based on the result of the Akaike information criterion (AIC) and Bayesian information criterion (BIC). ANOVA *F* statistic was performed to assess the potential non-linearity, and a line plot was used to visualize the results. The WQS analysis was performed with the *gWQS* package, and aging indicators were calculated with the *BioAge* package in R software (version 4.1.1). The Holm–Bonferroni correction was used to perform multiple comparisons for each PAH variable. A two-sided *P-*value of < 0.05 was considered to be statistically significant.

## 3 Results

### 3.1 Baseline characteristics

A total of 8,100 participants aged 12–85 years (mean age, 40.9 years) were included in this study. Table 1 summarizes the baseline characteristics of the study population by age quartile categories. Compared with low-age participants (mean age, 15.7 years old), participants with a mean age of 70.9 years were more likely to be educated at the high school level or less, overweight, physically inactive, never smokers, drinkers, and have more comorbidities such as diabetes or hypertension (all *P* < 0.001). At baseline, Supplementary Figure S1 shows that both KDM-BA (r = 0.962, *P* = 2.2 × 10^−16^) and PhenoAge (r = 0.971, *P* = 2.2 × 10^−16^) are positively correlated with chronological age.

**Table 1:** Baseline characteristics of participants by age in the NHANES datasets.

**Variables**	**Age (quartile)**				
	**Q1**	**Q2**	**Q3**	**Q4**	***P***
Age (years, mean ± SD)	15.7 ± 2.4	29.7 ± 5.6	49.0 ± 5.6	70.9 ± 7.4	< 0.001
Sex (*n* [%])					0.001
Male	1,087 (51.9)	945 (46.1)	991 (50.6)	1,004 (50.3)	
Female	1,008 (48.1)	1,106 (53.9)	968 (49.4)	991 (49.7)	
Race (*n* [%])					< 0.001
Mexican American	607 (29.0)	467 (22.8)	368 (18.8)	313 (15.7)	
Other Hispanic	149 (7.1)	176 (8.6)	140 (7.1)	106 (5.3)	
Non-Hispanic white	616 (29.4)	891 (43.4)	967 (49.4)	1,197 (60.0)	
Non-Hispanic black	629 (30.0)	395 (19.3	394 (20.1)	323 (16.2)	
Other race	94 (4.5)	122 (5.9)	90 (4.6)	56 (2.8)	
Education (*n* [%])					< 0.001
High school or less	61 (2.9)	985 (48.0)	976 (49.8)	1,207 (60.5)	
Some college	39 (1.9)	644 (31.4)	559 (28.5)	420 (21.1)	
College graduate or above	1 (0.1)	422 (20.6)	420 (21.5)	366 (18.3)	
Missing value	1,994 (95.1)	0 (0)	4 (0.2)	2 (0.1)	
BMI (kg/m^2^, mean± SD)	24.2 ± 6.3	28.4 ± 6.7	29.4 ± 7.0	28.8 ± 5.7	< 0.001
BMI (kg/m^2^, *n* [%])					< 0.001
Normal (< 25)	1,368 (65.3)	712 (34.7)	511 (26.1)	501 (25.1)	
Overweight (25 to 29.9)	396 (18.9)	646 (31.5)	669 (34.2)	756 (37.9)	
Obesity (≥30)	314 (15.0)	684 (33.4)	766 (39.1)	709 (35.5)	
Missing value	17 (0.8)	9 (0.4)	13 (0.6)	29 (1.5)	
Activity (*n* [%])					< 0.001
No or low	627 (29.9)	856 (41.7)	908 (46.3)	1,144 (57.3)	
Moderate	396 (18.9)	519 (25.3)	517 (26.4)	562 (28.2)	
Vigorous	1,034 (49.4)	670 (32.7)	515 (26.3)	237 (11.9)	
Missing value	38 (1.8)	6 (0.3)	19 (1.0)	52 (2.6)	
PIR (*n* [%])					< 0.001
< 1	636 (30.4)	471 (23.0)	343 (17.5)	264 (13.2)	
≥1	1,358 (64.8)	1,444 (70.4)	1,489 (76.0)	1,550 (77.7)	
Missing value	101 (4.8)	136 (6.6)	127 (6.5)	181 (9.1)	
Serum cotinine (*n* [%])					< 0.001
< LOD	359 (17.2)	326 (15.9)	343 (17.5)	516 (25.9)	
LOD-10	1,431 (68.3)	1,059 (51.6)	1,001 (51.1)	1,143 (57.3)	
>10	302 (14.4)	666 (32.5)	614 (31.3)	333 (16.7)	
Missing value	3 (0.1)	0 (0)	1 (0.1)	3 (0.1)	
Alcohol drinking (*n* [%])					< 0.001
No	37 (1.8)	449 (21.9)	453 (23.1)	675 (33.8)	
Yes	61 (2.9)	1,450 (70.7)	1,389 (70.9)	1,233 (61.8)	
Missing value	1,997 (95.3)	152 (7.4)	117 (6.0)	87 (4.4)	
Creatinine, urine (mg/dL)	164.6 ± 89.1	142.5 ± 84.6	128.0 ± 79.4	107.6 ± 68.4	< 0.001
Diabetes (*n* [%])					< 0.001
No	2,081 (99.3)	2,007 (97.9)	1,763 (90.0)	1,546 (77.5)	
Yes	14 (0.7)	44 (2.1)	196 (10.0)	449 (22.5)	
Hypertension (*n* [%])					< 0.001
No	1,998 (95.4)	1,577 (76.9)	1,191 (60.8)	566 (28.4)	
Yes	54 (2.6)	286 (13.9)	765 (39.1)	1,429 (71.6)	
Missing value	43 (2.0)	188 (9.2)	3 (0.1)	0 (0)	
1-Naphthol (ng/L, median [IQR])	1,696 (791, 4,373)	2,145 (905, 8,061)	2,663 (936, 9,998)	2,204 (929, 6,555)	< 0.001
2-Naphthol (ng/L, median [IQR])	3,922 (2,062, 7,665)	4,884 (2,100, 11,433)	4,473 (1,938, 10,486)	2,629 (1,219, 5,962)	< 0.001
3-Fluorene (ng/L, median [IQR])	108 (57, 217)	118 (50, 429)	107 (48, 424)	58 (32, 144)	< 0.001
2-Fluorene (ng/L, median [IQR])	276 (152, 516)	331 (150, 874)	309 (145, 880)	182 (98, 414)	< 0.001
1-Phenanthrene (ng/L, median [IQR])	144 (80, 262)	163 (89, 277)	163 (86, 293)	118 (64, 226)	< 0.001
1-Pyrene (ng/L, median [IQR])	130 (71, 257)	133 (66, 278)	111 (55, 244)	60 (30, 127)	< 0.001

Data were presented as mean ± SD, numbers and (proportions). Continuous variables were compared by one-way ANOVA Tests and categorical variables were compared by Chi-square tests. NHANES, National Health and Nutrition Examination Survey; SD, standard deviation; BMI, body mass index; PIR, Poverty income ratio; LOD, limit of detection; IQR, interquartile range.

### 3.2 Association between urinary PAHs and KDM-BA acceleration and PhenoAge acceleration

**Figure 2:**
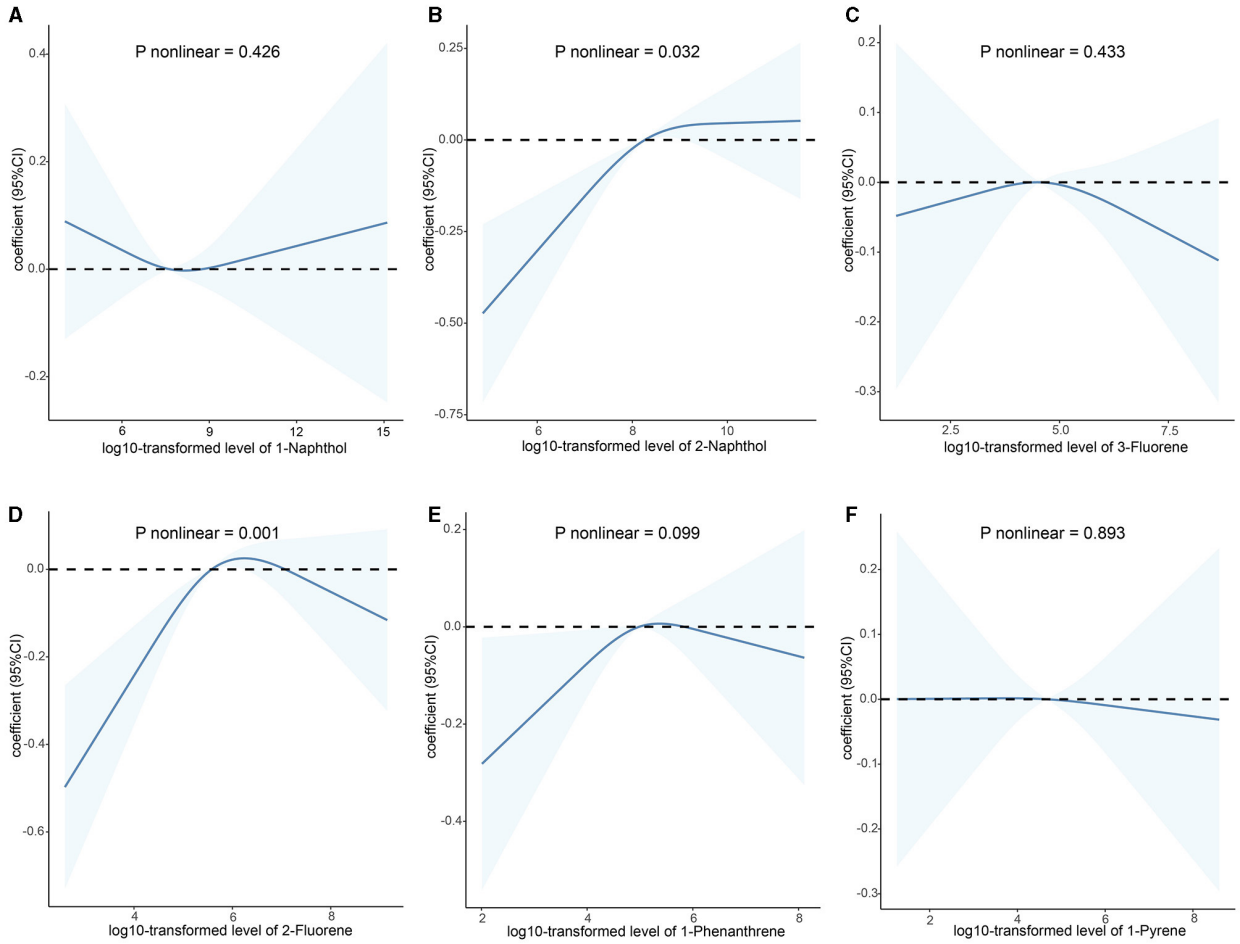
Association between urinary polycyclic aromatic hydrocarbons and KDM-BA acceleration based on the RCS analysis. **(A)** 1-Naphthol; **(B)** 2-Naphthol; **(C)** 3-Fluorene; **(D)** 2-Fluorene; **(E)** 1-Phenanthrene; **(F)** 1-Pyrene. The relationships were examined using multivariate ordinary least squares (OLS) regression models based on restricted cubic spline (RCS) analysis. The solid lines represent the estimates of effects and the dashed line represents the 95% CIs. The model was adjusted for age, sex, race, education level, activity, BMI, PIR, serum cotinine, alcohol consumption, creatinine, NHANES cycle, diabetes, and hypertension. RCS, restricted cubic splines; CI, confidence interval; KDM-BA, Klemera–Doubal Method—Biological Age.

**Figure 3:**
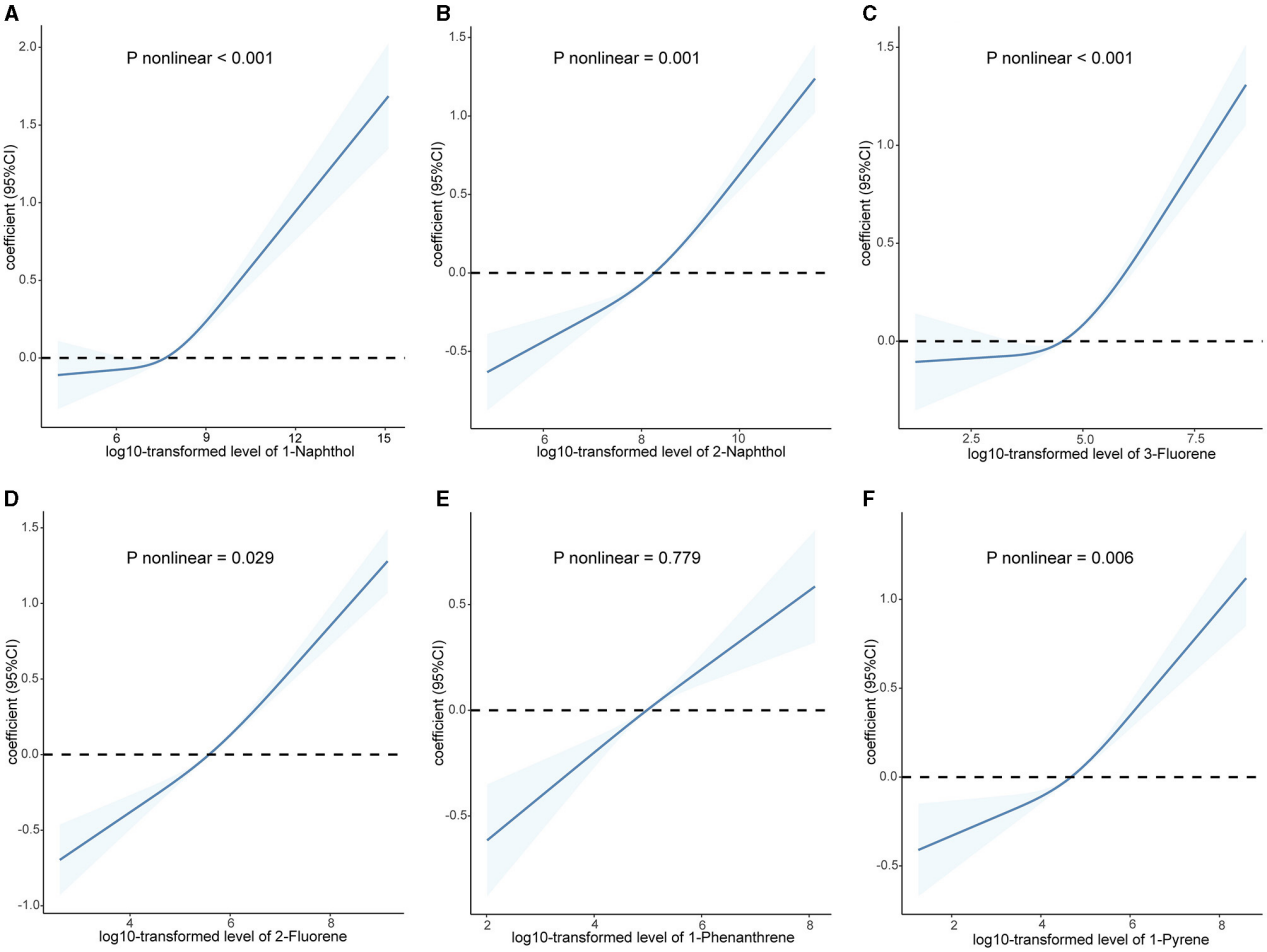
Association between urinary polycyclic aromatic hydrocarbons and PhenoAge acceleration based on the RCS analysis. **(A)** 1-Naphthol; **(B)** 2-Naphthol; **(C)** 3-Fluorene; **(D)** 2-Fluorene; **(E)** 1-Phenanthrene; **(F)** 1-Pyrene. The relationships were examined using multivariate ordinary least squares (OLS) regression models based on restricted cubic spline (RCS) analysis. The solid lines represent the estimates of effects and the dashed line represents 95% CIs. The model was adjusted for age, sex, race, education level, activity, BMI, PIR, serum cotinine, alcohol consumption, creatinine, NHANES cycle, diabetes, and hypertension. RCS, restricted cubic splines; CI, confidence interval.

As shown in Supplementary Figure S2, the majority of urinary PAHs are associated with each other except for 1-naphthol and 1-phenanthrene or 1-pyrene, ranging from moderate to high correlation (Spearman's r = 0.03–0.93). Table 2 presents the relationship between urinary PAHs and KDM-BA acceleration and PhenoAge acceleration. After adjusting for age, sex, race, education level, activity, BMI, PIR, smoking status, alcohol consumption, creatinine, NHANES cycle, diabetes, and hypertension, each unit increase in the log10-transformed level of 1-phenanthrene is associated with a 0.191 (95% CI: −0.331, −0.050) decrease in KDM-BA acceleration. However, these relationships were not statistically significant after a Holm–Bonferroni correction. After the same multiple variable adjustment, each unit increase in the log10-transformed level of 1-naphthol, 2-naphthol, 3-fluorene, and 2-fluorene is associated with a 0.173 (95% CI: 0.085, 0.261), 0.310 (95% CI: 0.182, 0.438), 0.159 (95% CI: 0.026, 0.292), and 0.454 (95% CI: 0.309, 0.598) -year increase in PhenoAge acceleration, respectively. After Holm–Bonferroni multiple corrections, these associations remain statistically significant except for 3-fluorene (all corrected *P* < 0.05). The results of RCS present a non-linear association between 2-naphthol (*P* = 0.032) and 2-fluorene (*P* = 0.001) and KDM-BA acceleration (Figure 2). In addition, we observed a J-shaped curve association between 1-naphthol (*P* < 0.001), 2-naphthol (*P* = 0.001), 3-fluorene (*P* < 0.001), 2-fluorene (*P* = 0.029), and 1-pyrene (*P* = 0.006) with PhenoAge acceleration (Figure 3). The results of the subgroup analysis are listed in Supplementary Table S2. We observed that the effect of 1-naphthol is more significant in the age (≥60 year) and male group (all *P* < 0.05); the effect of 2-naphthol is more obvious in the < 60-year-old and male group (All *P* < 0.05); and the effect of 2-fluorene is more obvious in the < 60-year-old and male group (all *P* < 0.05).

**Table 2:** Association of urinary polycyclic aromatic hydrocarbons and aging indexes.

**PAH**	**KDM-BA acceleration**		**PhenoAge acceleration**	
	β **(95% CI)**	**P**	β **(95% CI)**	***P***
1-Naphthol	0.007 (−0.884, 0.102)	0.892	0.173 (0.085, 0.261)	**1.1** **×10**^**−04**^
2-Naphthol	0.097 (−0.041, 0.236)	0.168	0.310 (0.182, 0.438)	**2.2** **×10**^**−06**^
3-Fluorene	−0.102 (−0.245, 0.041)	0.163	0.159 (0.026, 0.292)	0.019
2-Fluorene	0.166 (0.010, 0.322)	0.038	0.454 (0.309, 0.598)	**8.3** **×10**^**−10**^
1-Phenanthrene	−0.132 (−0.295, 0.031)	0.112	0.128 (−0.023, 0.279)	0.097
1-Pyrene	−0.191 (−0.331, −0.050)	0.008	0.077 (−0.054, 0.207)	0.250

The urinary concentrations of 6 polycyclic aromatic hydrocarbons (ng/L) were log-transformed in the regression models. The log-transformed level of each PAH was separately included in the single-exposure models, with adjusted for age (continuous, year), sex (male, female), race (Mexican American, Other Hispanic, Non-Hispanic White, Non-Hispanic Black, Other Race), education level (high school or less, some college, college graduate or above, missing), activity (no or low, moderate, vigorous, missing), body mass index (< 25 kg/m^2^, 25 to 29.9 kg/m^2^, ≥30 kg/m^2^, missing), poverty income ratio (< 1, ≥1, missing), serum cotinine (< LOD, LOD-10, >10, missing), alcohol consumption (no, yes, missing), creatinine (continuous, mg/dL), NHANES cycle (2003–2004, 2005–2006, 2007–2008, and 2009–2010), diabetes (yes, no) and hypertension (yes, no, missing). Bolded values indicate statistically significant associations at Holm-Bonferroni correction < 0.008 (0.05/6). PAH, polycyclic aromatic hydrocarbon; KDM-BA, Klemera Double method Biological Age; CI, confidence interval; LOD, limit of detection.

### 3.3 Mixture effects of multiple PAHs on KDM-BA acceleration and PhenoAge acceleration

**Figure 4:**
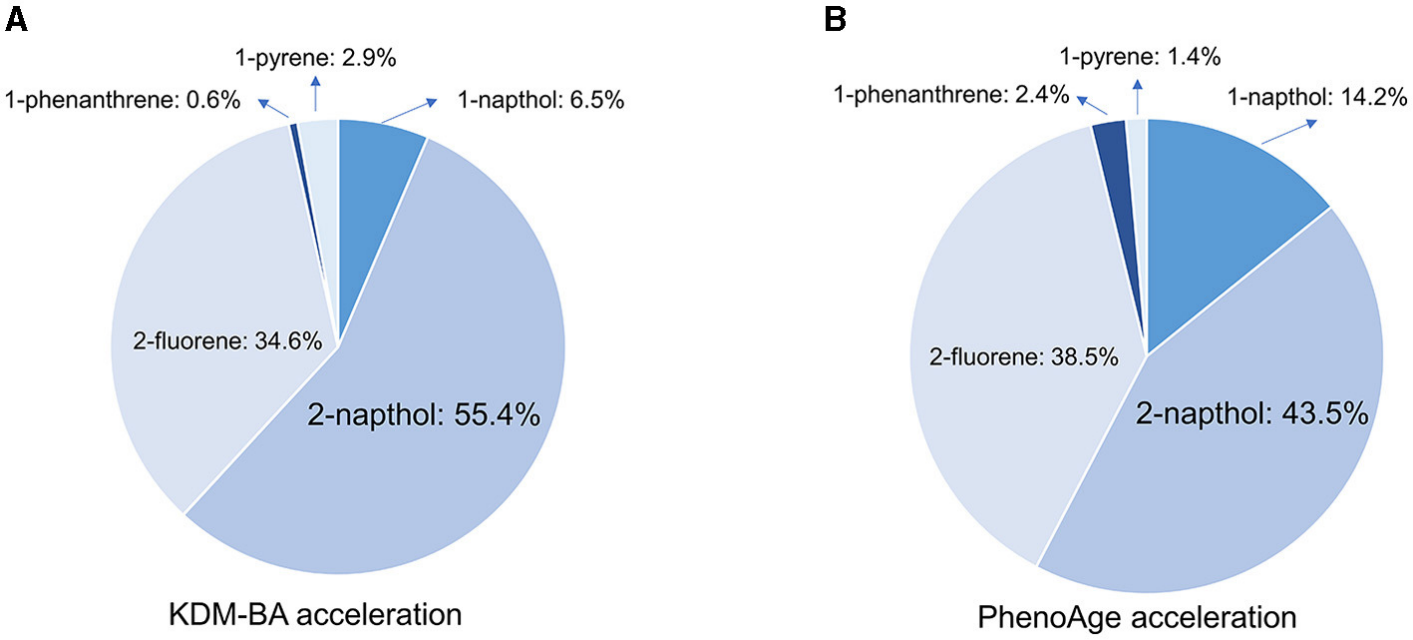
Weighted quantile sum (WQS) model regression index weights for KDM-BA and PhenoAge in adults. **(A)** KDM-BA; **(B)** PhenoAge. Models were adjusted for age, sex, race, education level, activity, BMI, PIR, serum cotinine, alcohol consumption, creatinine, NHANES cycle, diabetes, and hypertension.

In the fully adjusted WQS regression model, the WQS index was negatively associated with KDM-BA acceleration (β[95%CI]: 0.13[0, 0.26], *P* = 0.048) and PhenoAge acceleration (β[95%CI]: 0.59[0.47, 0.70], *P* < 0.001). The estimated PAH metabolite weights of KDM-BA and PhenoAge are presented in Figure 4. The highest weighted PAH metabolite in both the KDM-BA acceleration and PhenoAge acceleration was 2-naphthol (weighted 55.4% in the KDM-BA acceleration and 43.5% in the PhenoAge acceleration, respectively).

## 4 Discussion

Our results in the present study showed that individual and mixed exposure to PAHs is positively associated with biological age, with 2-naphthol contributing the most, and 2-naphthol this PAH has a dose–response relationship with biological age indicators.

Our results revealed that increased PAH exposure is related to an increase in biological age, and each unit increase in the log10-transformed level of 1-naphthol, 2-naphthol, 3-fluorene, and 2-fluorene is associated with a 0.173-, 0.310-, 0.159- and 0.454-year increase in PhenoAge acceleration, leading to the suggestion that increased PAH concentration may cause aging. Our results are in conflict with previous evidence regarding PAHs causing aging. Li et al. found that PAH exposure was positively associated with DNA methylation aging in 1,149 subjects. The result suggested that every one unit increase in 1-hydroxypyrene and 9-hydroxyphenanthrene was associated with a 0.53- and 0.54-year increase in aging markers (2). Another research showed that every one unit increase in the sum of 1- and 2-hydroxynaphthalene was associated with a 0.37-year increase in PhenoAge acceleration in 2,579 subjects (21). To date, these studies are the only reports of epidemiological studies on PAHs and aging. The difference between our research and the other studies mentioned is that we have found for the first time a correlation between 1-naphthol, 2-naphthol, 3-fluorene, and 2-fluorene and aging indicators. In addition, we also found for the first time that exposure to mixed PAHs is associated with aging indicators. Mixed exposure often better reflects real-world exposure and identifies risk factors for diseases compared to individual exposure (22). Previous publications have also demonstrated that mixed exposure to PAHs may increase the incidence of adverse health outcomes, such as metabolic syndrome (23), liver function (24, 25), hypertension, and lung function (26). Our study further expands the hazards of mixed exposure to PAHs and suggests a correlation between PAHs and aging.

Although we found an association between PAHs and aging in this study, as our study design was cross-sectional, we cannot directly demonstrate the role of PAHs in aging. However, previous research has provided biological evidence for PAH-induced aging. Aging is characterized by telomere dysfunction, oncogene activation, and sustained DNA damage (27). First, PAHs were found to cause telomere dysfunction during aging and apoptosis in male germ cells (28). Additionally, PAHs have been regarded as carcinogens (29), with the ability to activate oncogenes. Moreover, PAH–DNA adduct formation has been reported in previous studies (30, 31), providing evidence that PAHs cause DNA damage. These previous reports have demonstrated that PAHs can induce molecular mechanisms that drive the aging process. This body of evidence from previous reports suggests that PAHs may cause aging in terms of molecular biology.

Our research findings may have public health implications. At present, our understanding of aging is insufficient. To reduce the occurrence of aging at the population level, the premise is to clarify the causes and risk factors of aging. Previous studies have pointed out that genetics and lifestyle are the main risk factors for aging (32, 33). In this study, we found that high exposure to PAHs is also a risk factor for increased aging. Further research is needed to confirm that PAHs are a risk factor for aging in the future. Our findings suggest that one of the possible clues to slow down aging is to implement possible measures to reduce PAH exposure. At present, the main pathways of human exposure to PAHs are anthropogenic pollution sources, including industrial, mobile, domestic, and agricultural pollution sources (34, 35). Therefore, staying away from PAH exposure sources can reduce risks; in addition, PAH concentrations in environmental media need to be reduced through physical, chemical, and biological PAH remediation (36). In addition, personal protection measures against PAHs can also be increased, which can effectively reduce PAH exposure levels and ultimately delay the aging caused by PAHs at the population level. In addition, we can provide a reference for the mechanism study of PAH exposure and disease. For example, Duan et al. found that exposure to PAHs may increase the risk of cardiovascular disease, mortality, all-cause mortality, and the mediation role of phenotypic aging (37).

Our research has the following advantages: First, the biological age, an indicator of aging, is based on 12 clinical indicators and calculations. This indicator is relatively easy to detect compared with previous markers, such as telomerase activity, telomere length, DNA methylation, and mitochondrial DNA content, which is convenient for large-scale popularization in the population. Second, our study revealed for the first time the associations between mixed PAH exposure and aging, which supplements the impact of mixed PAH exposure on aging. Third, based on the mixed exposure model, we observed that 2-naphthol has the greatest impact on the association between PAH components and aging. This result suggests that 2-naphthol is a sensitive chemical in the impact of PAHs on aging.

At the same time, the present study has the following limitations. First, this study is a cross-sectional study, and due to the nature of the study design, it is not possible to explain causal relationships. For such limitations, we provided biological results for the causal evidence of PAHs and aging in the Discussion section. Second, the covariate adjustment carried out in this study is still insufficient, as the impact of genetics on aging cannot be ignored. However, due to the lack of genetic information in NHANES, this covariate cannot be included in this study, and future research needs to consider this factor. In addition, the underlying mechanisms of PAH exposure and aging should be further investigated in *in vivo/vitro* experiments.

## 5 Conclusion

Exposure to mixed PAH is associated with increased aging, with 2-naphthol being the largest contributor among PAH components. This study identified risk factors among PAH components for aging.

## Data availability statement

The datasets presented in this study can be found in online repositories. The names of the repository/repositories and accession number(s) can be found in the article/Supplementary material.

## Ethics statement

The studies involving humans were approved by the CDC/NCHS Research Ethics Review Board. The studies were conducted in accordance with the local legislation and institutional requirements. The participants provided their written informed consent to participate in this study.

## Author contributions

ZF: Conceptualization, Data curation, Investigation, Supervision, Validation, Visualization, Writing – original draft, Writing – review & editing. XZ: Investigation, Methodology, Writing – original draft. CZ: Investigation, Methodology, Writing – review & editing. ZG: Investigation, Methodology, Writing – review & editing. QY: Conceptualization, Data curation, Investigation, Methodology, Supervision, Validation, Visualization, Writing – review & editing, Writing – original draft.
