# Supplementary material for: Association between single and mixed exposure to polycyclic aromatic hydrocarbons and biological aging
Source: Front Public Health. 2024 Jun 5;12:1379252. doi: 10.3389/fpubh.2024.1379252 (PMC11188445; doi:10.3389/fpubh.2024.1379252)
Supplement: Supplementary file 1 [file Data_Sheet_1.docx]

**Supplementary materials**

**Association between mixed exposure of** **polycyclic aromatic hydrocarbon and biological aging**

Zuqiang Fu, Xianli Zhang, Chunyu Zhong, Zhe Gao, Qing Yan

**Contents**

**Supplementary tables**

**Table S1.** The limits of detection and distribution of urinary polycyclic aromatic hydrocarbons**.**

**Table S2.** Subgroup analyses for the associations of urinary PAH1, PAH2 and PAH4 with PhenoAge acceleration by age and sex.

**Supplementary figures**

**Figure S1**. **Correlation matrix of chronological age, biological ages, and age accelerations.** (A) KDM-BA acceleration; (B) PhenoAge acceleration. Abbreviations: KDM-BA, Klemera Double method Biological Age.

**Figure S2**. **Spearman’s correlation coefficients for concentrations of 6 urinary PAHs.** The color square box represents the size of the correlation, and the center value is “r”. “×” means the correlation is not statistically significant. Abbreviations: PAH, polycyclic aromatic hydrocarbon.

**Table S1.** The limits of detection and distribution of urinary polycyclic aromatic hydrocarbons

| PAH (ng/L) | N | Medium [IQR] | LOD | Above LOD (%) |
| --- | --- | --- | --- | --- |
| 1-Naphthol | 7,205 | 2081.2 (879.9, 6904.7) | 60 | 99.9 |
| 2-Naphthol | 7,488 | 3874.0 (1774.9, 8912.4) | 90 | 99.9 |
| 3-Fluorene | 3,592 | 94.3 (45.2, 267) | 8 | 99.4 |
| 2-Fluorene | 4,914 | 265.0 (131.1, 624.0) | 8 | 100.0 |
| 1-Phenanthrene | 3,554 | 145.7 (79.0, 265.0) | 9 | 99.9 |
| 1-Pyrene | 3,334 | 107.3 (52.0, 227.9) | 10 | 99.5 |

Abbreviations: PAH, polycyclic aromatic hydrocarbon; LOD, limits of detection

**Table S2.** Subgroup analyses for the associations of urinary PAH1, PAH2 and PAH4 with PhenoAge acceleration by age and sex.

| Subgroup | n (%) | β (95% CI) | P |
| --- | --- | --- | --- |
| 1-Naphthol | | | |
| Age (years) |  |  |  |
| <60 | 6,105 (75.4) | 0.140 (0.043, 0.237) | 0.005 |
| ≥60 | 1,995 (24.6) | 0.255 (0.062, 0.447) | 0.01 |
| Sex |  |  |  |
| Male | 4,027 (49.7) | 0.180 (0.059, 0.301) | 0.004 |
| Female | 4,073 (50.3) | 0.133 (0.007, 0.259) | 0.039 |
| 2-Naphthol | | | |
| Age (years) |  |  |  |
| <60 | 6,105 (75.4) | 0.395 (0.255, 0.535) | <0.001 |
| ≥60 | 1,995 (24.6) | 0.018 (-0.277, 0.313) | 0.904 |
| Sex |  |  |  |
| Male | 4,027 (49.7) | 0.317 (0.145, 0.489) | <0.001 |
| Female | 4,073 (50.3) | 0.208 (0.017, 0.400) | 0.033 |
| 2-Fluorene | | | |
| Age (years) |  |  |  |
| <60 | 6,105 (75.4) | 0.519 (0.361, 0.676) | <0.001 |
| ≥60 | 1,995 (24.6) | 0.260 (-0.079, 0.599) | 0.133 |
| Sex |  |  |  |
| Male | 4,027 (49.7) | 0.448 (0.260, 0.636) | <0.001 |
| Female | 4,073 (50.3) | 0.334 (0.109, 0.559) | 0.004 |

Multiple linear regression models, with adjustments for age, sex, race, education level, activity, BMI, PIR, serum cotinine, alcohol consumption, creatinine, NHANES cycle, diabetes and hypertension, except for the stratification variable;

PAH1 represented 1-Naphthol, PAH2 represented 2-Naphthol, and PAH4 represented 2-Fluorene in sequence.

Abbreviations: PAH, polycyclic aromatic hydrocarbon; CI, confidence interval; PIR: Poverty income ratio; BMI, body mass index.


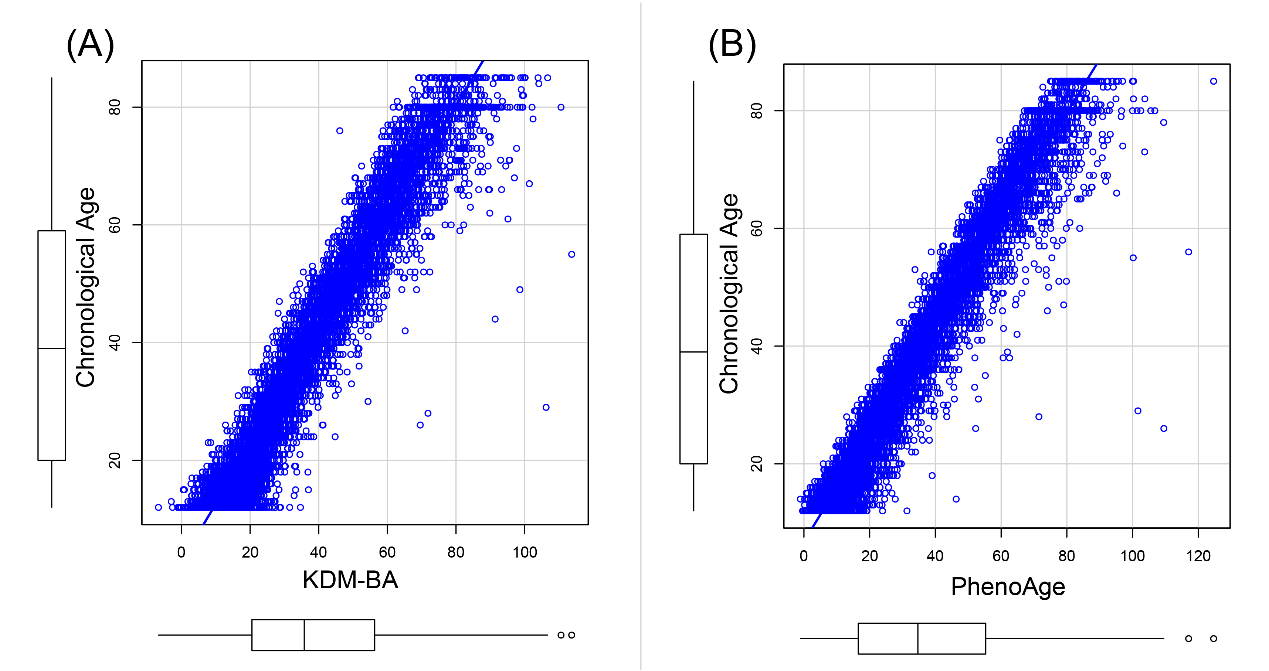


**Figure S1. Correlation matrix of chronological age, biological ages, and age accelerations.** (A) KDM-BA acceleration; (B) PhenoAge acceleration. Abbreviations: KDM-BA, Klemera Double method Biological Age.


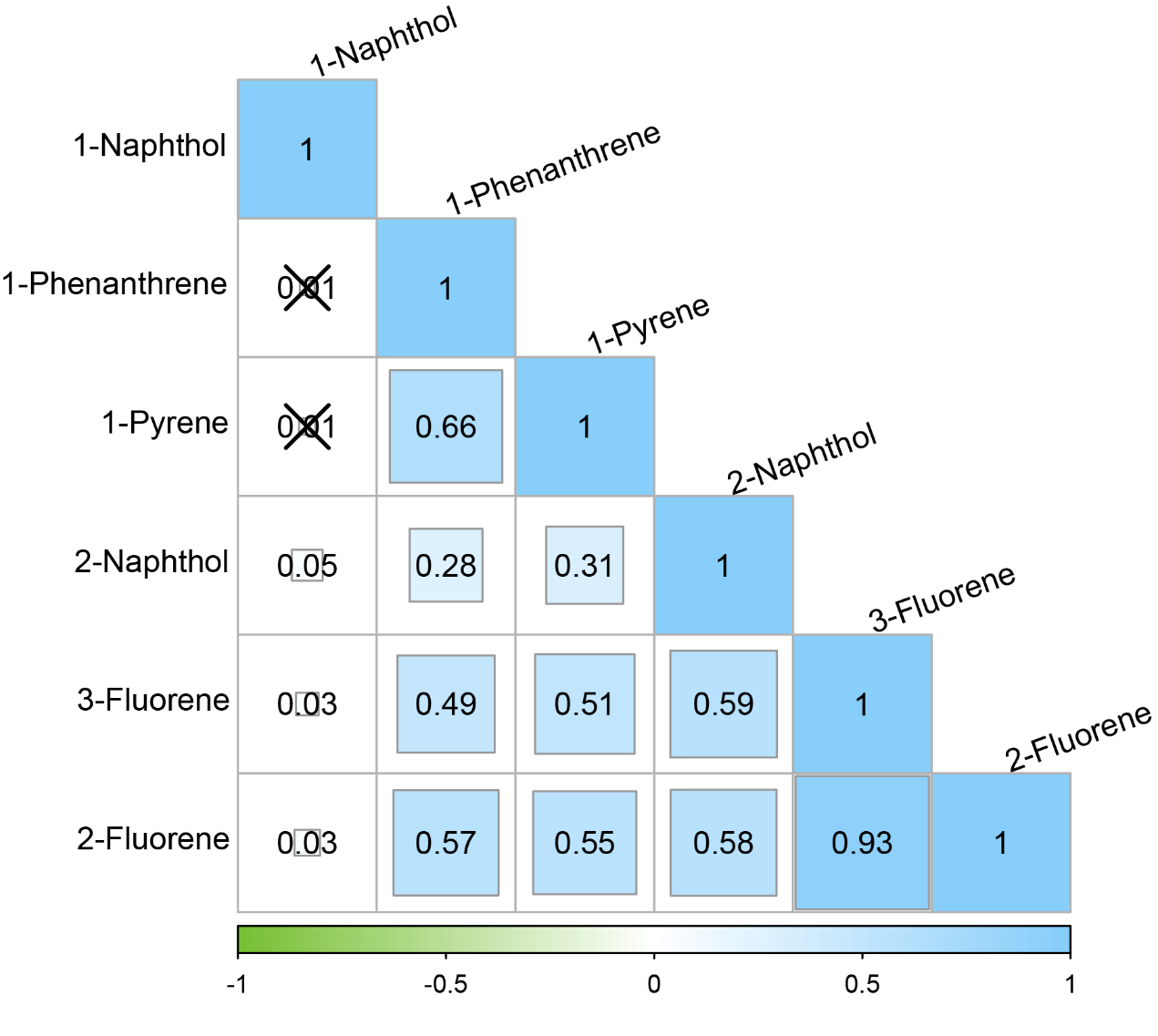


**Figure S2. Spearman’s correlation coefficients for concentrations of 6 urinary PAHs.** The color square box represents the size of the correlation, and the center value is “r”. “×” means the correlation is not statistically significant.

Abbreviations: PAH, polycyclic aromatic hydrocarbon.
